# Milk Somatic Cell Count and Polymorphonuclear Cells in Healthy Quarters of Cows That Underwent Blanket and Selective Dry Therapy: An Italian Case Study

**DOI:** 10.3390/vetsci8120298

**Published:** 2021-11-29

**Authors:** Angela Costa, Massimo De Marchi, Daniele Sagrafoli, Hillary Lanzi, Simonetta Amatiste, Carlo Boselli, Giuseppina Giacinti

**Affiliations:** 1 Department of Agronomy, Food, Natural Resources, Animals and Environment (DAFNAE), University of Padova, Viale dell’Università 16, 35020 Legnaro, Italy; massimo.demarchi@unipd.it; 2 Experimental Zooprophylactic Institute Lazio and Toscana Mariano Aleandri, Via Appia Nuova 1411, 00178 Rome, Italy; daniele.sagrafoli@izslt.it (D.S.); simonetta.amatiste@izslt.it (S.A.); carlo.boselli@izslt.it (C.B.); giuseppina.giacinti@izslt.it (G.G.); 3 Private Practitioner, Via San Benedetto, 26, 03010 Torre Cajetani, Italy; hillarylanzi1@gmail.com

**Keywords:** drying off, mastitis, udder health, differential somatic cells, neutrophils

## Abstract

The incidence of mastitis increases with parity in dairy cattle and multiparous cows are often treated at drying off to limit the risk of udder health issues and support mammary gland tissues recovery. Milk somatic cells count (SCC, cells/mL) comprises different white blood cells fractions and is worldwide used to monitor and genetically improve udder health. Nevertheless, only certain SCC fractions increase when an udder inflammation occurs. Considering that antibiotic use for preventive purposes will be forbidden in 2022, we compared two different dry therapy protocols, blanket (BDCT) and selective (SDCT), on different SCC fractions in healthy quarters milk. Multiparous Holstein cows were enrolled in a randomized controlled trial and SCC, neutrophils, macrophages, lymphocytes, polymorphonuclear cells (PMN) and differential somatic cell count (DSCC) recorded after the experimental drying off were available. Significant differences were observed between the two protocols, with more favorable parameters in BDCT than SDCT cows. Results showed that moving from BDCT to SDCT is expected to significantly increase some SCC fractions, such as PMN, in healthy quarters. The baseline SCC level at the onset of lactation was greater in cows under SDCT than BDCT. Although not significant, clinical mastitis prevalence was numerically lower in BDCT (7.32%) than SDCT (8.62%). In this study we referred to a limited number of cows, but still findings will be useful to improve the knowledge on the impact of SDCT on milk SCC fractions in healthy quarters.

## 1. Introduction

Mastitis continues to be the most expensive disease in dairy cattle worldwide, causing direct and indirect economic losses at farm level, such as discarded milk, impaired productivity, and veterinarian treatments [1,2]. In cosmopolitan dairy breeds, both mastitis incidence and level of milk somatic cell count (SCC, cells/mL) increase with parity [1,2,3] and farmers use to administer therapeutic treatments during the dry period in order to reduce the risk of mastitis in the subsequent lactation and limit proliferation of pathogens in the udder environment during non-productive periods. In fact, the dry period can also be considered as an intermediate stage intended for the mammary gland tissues recovery and renewal of the alveolar epithelium [4]. 

Several schools of thought on the approach to dry cow therapy (DCT) coexist, i.e., blanket, selective and absent DCT [4,5]. Herds adopting blanket DCT (BDCT) are subjected to specific costs for the preventive treatment on all cows and quarters regardless of the health status and/or the SCC level before drying off. Overall, it has been demonstrated that BDCT reduces the risk of mastitis in the subsequent lactation as well as the risk of having milk SCC greater than 200,000 cells/mL, the conventional alert threshold, compared to selective and absent DCT [6]. In order to avoid preventive treatments, reduce economic inputs, and limit antimicrobial use on farm, the selective DCT (SDCT) has been proposed. In fact, considering the upcoming European legislation on the antimicrobial use, preventive treatments in livestock will be forbidden in 2022. Based on recent studies, when SDCT is properly implemented it is cost-effective and could not impair herd udder health in the long-term [7,8]. However, various SDCT protocols exist and significantly differ in terms of labor and effectiveness [6]. The idea behind SDCT is that only the most susceptible quarters or cows have to be treated with antibiotic before drying off, e.g. quarters presenting milk SCC at the last test-day above a certain threshold [6] and/or those positive at the California Mastitis Test [5]. In this regard, the criteria used to assign SDCT to a quarter or a cow differs among studies, as widely reviewed by [5]. Selection of quarters or cows to be treated depends also on herd-specific SCC baseline level, breed, season, and other cow-independent factors [4,5,6,7,8]. Generally, the most popular SDCT protocols aims at treating quarters presenting milk SCC >200,000 cells/mL at the last test-day, i.e., in late lactation [6]. Several research articles investigated the effect of DCT on healthy quarters milk SCC, but without looking at differences in detailed SCC fractions, namely macrophages, neutrophils, and lymphocytes. A better understanding of changes in SCC fractions is important, as it has been demonstrated that only neutrophils and lymphocytes are elevated in high-SCC milk and/or in mastitic milk [9,10,11]. Compared to the conventional total SCC, neutrophils and lymphocytes could be more informative of the mammary gland health status [1]. In addition, although milk SCC had showed a decreasing trend in dairy cows worldwide, still mastitis is a widespread problem in both its clinical and subclinical form [12].

The aim of the present study was to compare two dry period protocols, traditional BDCT and alternative SDCT, on healthy quarters milk by looking at differences after drying off in total SCC and SCC fractions.

## 2. Materials and Methods

### 2.1. Experimental Groups and Inclusion Criteria 

The experiment took place from January to June 2017 in a commercial purebred Holstein dairy farm located in Central Italy (Maccarese S.p.A., Fiumicino, Italy) and was a blind randomized trial with farm personnel blinded to the DCT. The study was in accordance with published guidelines for randomized control trials for livestock and food safety [13]. The present study was funded and approved by the Italian Minister of Health (Rome, Italy) and no ethic approval was required, as two dry period protocols commonly available were compared. The official veterinarian of the farm was in charge of the antibiotic treatments on the cows and a written informed consent was obtained from the owner of animals. 

The two experimental groups were randomly defined in advance (December 2016) according to tag ID number by considering the list of cows expected to undergo drying off in the observing period. In particular, even and odd digits of cows’ tag ID number were considered for allocation to BDCT and SDCT, respectively. At the end of the study, in June 2017, the BDCT and the SDCT group were balanced in terms of number of cows. 

Briefly, the trial consisted in three different phases:

- Quarter milk collection and analysis 24 h before drying off;
- Administration of the assigned DCT to each cow;
- Quarter milk collection and analysis after the dry period.

Milk sampling at the end of the dry period took place on average 8.93 d after calving, with minimum and maximum of 7 and 14 d. However, only in few cases (7% of cows) the samples were collected later than 11 d after calving. In this regard, following conventional standards, quarter milk sampling took place some days after parturition to avoid collection of colostrum, a matrix with different characteristics compared to mature milk in terms of density and protein composition and content [14]. In fact, as reported by the International Committee for Animal Recording [15], mammary gland secretion of the first 4 d is usually not considered as mature milk. Overall, average, minimum, and maximum dry period length were 65, 37, and 81 d, respectively. 

The protocol adopted for BDCT cows included intra-mammary antibiotic treatments with 250 g cephazoline (Cefovet A, Merial Italia SpA, Milan, Italy) in all the quarters, regardless of SCC analysis. On the contrary, cows in the SDCT group were subjected to a quarter-level antibiotic treatment (Cefovet A, Merial Italia SpA, Milan, Italy) only in the affected and suspicious quarters. The latter were identified by using the Qscout Milk Leukocyte Differential test implemented in the QScout^®^ Farm Lab portable device (Advanced Animal Diagnostics, Inc., Morrisville, NC, USA). As extensively described by [16], this device is able to determine the SCC fractions and combine them for identification of suspicious (‘borderline’) and affected quarters (‘positive’) exploiting the algorithms installed. The Qscout Milk Leukocyte Differential test implemented in the QScout^®^ Farm Lab portable device (Advanced Animal Diagnostics, Inc., Morrisville, NC, USA) performs a discriminant analysis of the sample (‘negative’, ‘borderline’, or ‘positive’) and provides concentration (cells/mL) of total milk SCC, macrophages (M), and polymorphonuclear cells (PMN), namely neutrophils (N) and lymphocytes (L). Information on specificity and sensitivity of the device have been reported by the manufacturer and several authors [16,17].

Quarter milk was collected and analyzed according to the International Committee for Animal Recording (ICAR) guidelines both before and after the experimental dry period [15]. The microbiological analysis was carried out within 24 h from milk samples collection at the laboratory of the Experimental Zooprophylactic Institute Lazio and Toscana Mariano Aleandri (Rome, Italy). Isolation and identification of pathogens were performed following the POS CIP 005 protocol recommended by the National Mastitis Council [18]. Teat sealants (Orbeseal, Zoetis Italia S.r.l., Latina, Italy) were inserted in all the cows involved in the study, regardless of the experimental group. Cows of BDCT and SDCT were subjected to the same farming and feeding system for the whole dry period.

Out of the 210 cows originally enrolled, 10 were discarded due to presence of clinical mastitis and/or lameness diagnosis in the last 90 d of lactation. Moreover, since the purpose of the present study was to evaluate the effect of the two DCT on SCC-related traits of healthy quarters, we excluded all the cows presenting, before drying off, ≥1 quarter labelled either ‘positive’ or ‘borderline’ and/or ≥1 quarter positive to microbiological analysis. In other words, the affected or suspicious quarters that were treated with antibiotic under either SDCT or BDCT were excluded in order to keep the focus on healthy ones, ending up with 453 quarters of 169 cows. Out of these animals, 70 and 99 were linked to milk data of ≤2 and ≥3 functional and healthy quarters, respectively. 

In order to guarantee a minimum number of measurements per cow in the statistical analysis, the focus was put on the 99 cows with at least 3 healthy and functional quarters presenting a comprehensive milk composition with no missing or outlier values for SCC, M, N, L, and PMN (Table 1). In particular, 144 and 202 quarter milk records belonging to 41 and 58 cows were available for BDCT and SDCT, respectively. The two experimental groups were similar in terms of frequency across parity orders and average dry period length (Table 1).

### 2.2. Statistical Analysis

To compare BDCT and SDCT in terms of SCC traits, the concentration (cells × 10^3^/mL) of SCC, N, M, L, and PMN determined by the device was log-transformed in order to normalize data distribution, as follows:

- SCS = 3 + log_2_(SCC/100);
- N_S_ = log_2_(N);
- M_S_ = log_2_(M);
- L_S_ = log_2_(L);
- PMN_S_ = 3 + log_2_(PMN/100).

For each milk sample, the differential somatic cell count (DSCC, %) was calculated as the ratio of PMN to the total SCC.

The PROC TTEST of SAS software v. 9.4 (SAS Institute Inc., Cary, NC, USA) was adopted to understand comparability of the abovementioned traits in the two groups before the experimental dry period. In the same software, the PROC CORR was used to estimate Pearson correlations between the traits within the DCT, while the analysis of variance was performed with the PROC MIXED. In particular, least squares means (LSM) of fixed effects were estimated using the following linear mixed model accounting for repeated observation per cow and considering the nested design of the experiment:

*y_ijkl_* = *Month_i_* + *Group_j_* + *β(Test_ijkl_)* + *Cow_k_ (Group_j_)* + *e_ijkl_*,

where *y* is the vector of phenotypic observations of the dependent variable (SCS, N_S_, L_S_, M_S_, PMN_S_, or DSCC) recorded at quarter level after the experimental drying off, *Month* is the fixed effect of the i^th^ month of calving (January to June), *Group* is the fixed effect of the j^th^ experimental group (BDCT or SDCT), *Test* is a covariate for the fixed effect of the dependent variable at the last milk sampling before dry period with regression coefficient *β*, *Cow(Group)* is the random effect of the k^th^ cow nested within the experimental group, and *e* is the residual term. The Bonferroni post-hoc test was used for LSM multiple comparison. 

Based on [6], quarters presenting milk SCC ≥100,000 cells/mL after the experimental dry period were flagged as ‘high’ in order to calculate their prevalence (%) to the total quarters; this was calculated within the two experimental groups in order to compare BDCT and SDCT using the PROC MULTTEST with the Bonferroni–Holm step-down adjustment (SAS software v. 9.4, SAS Institute Inc., Cary, NC, USA). Exploiting the same approach, the prevalence (%) of quarters showing PMN ≥ 130,000 cells/mL after the experimental dry period were calculated for BDCT and SDCT and then compared. The present threshold, i.e., 130,000 cells/mL, corresponds to a DSCC of 0.65 when SCC is equal to 200,000 cells/mL; these are values conventionally used to discriminate healthy and suspicious cows (or quarters) with consensus at international level [19,20,21]. In a similar way, a comparison between BDCT and SDCT was performed for the prevalence (%) of quarters after drying off labelled as ‘positive’ or ‘borderline’ by the QScout^®^ Farm Lab software, for the prevalence (%) of quarters after drying off with ≥1 pathogen isolated, and for the prevalence (%) of clinical mastitis recorded between 0 and 305 d in milk after the experimental drying off. Each mastitis event was diagnosed by the official veterinarian and recorded in the farm management software.

## 3. Results

### 3.1. Data Overview

The QScout^®^ Farm Lab portable device (Advanced Animal Diagnostics, Inc., Morrisville, NC, USA) was used in this experimental trial to investigate the effect of two DCT protocols on some SCC-related traits. Descriptive statistics of the whole set of traits before and after the experimental DCT are reported in Table 2, separately for BDCT and SDCT. According to the statistical test, the parameters recorded before the experimental drying off were similar between groups, with the only exception of M_S_. The latter presented significantly greater average and standard deviation in SDCT (3.95 ± 1.36) than in BDCT quarters (3.58 ± 1.04). 

In both groups SCS tended to be greater and more variable after drying off than before, i.e., in milk sampled around calving. Milk SCC calculated by back-transforming SCS (Table 2) was on average around 45,000 and 80,000 cells/mL in BDCT and SDCT after drying off. As regards N_S_, M_S_, L_S_ and PMN_S_, both their mean and standard deviation were slightly greater after than before the dry period, regardless of the DCT (Table 2). On the other hand, the DSCC was similar in milk sampled before and after drying off (Table 2). In the whole dataset, the maximum N_S_, M_S_, and L_S_ observed after the experimental dry period was 13.64, 11.23, and 10.51 which approximately corresponds to a back-transformed concentration ×10^3^ cells/mL) of 12,766, 2402, and 1458, respectively. In a small number of quarters, the concentration of N (*n* = 30), M (*n* = 63), or L (*n* = 46) determined by the device was under the limit of detection and thus reported as equal to 0 cells/mL; for such quarters, N_S_, M_S_, or L_S_ were considered as missing. 

Pearson correlations were estimated separately for BDCT and SDCT using traits recorded after drying off (Table 3). Overall, correlations of BDCT resembled those calculated for SDCT; in particular, the strongest associations (0.97) in both cases were those calculated between SCS and PMN_S_ and between N_S_ and PMN_S_ (Table 3). All the other features were positively correlated to each other, with the exception of DSCC whose correlations were in certain cases not significant and/or negative (Table 3). In both DCT, the correlation between M_S_ and DSCC moved in the negative direction.

### 3.2. Analysis of Variance

Both month of calving and value of the trait at the last milk sampling before dry period were not significant in explaining the variability of the investigated traits. The only exception was PMN_S_, whose value at the last milk sampling before drying off had a significant effect (*p* = 0.004) on the value recorded after drying off.

A summary of the DCT effect is shown in Table 4, where the LSM of BDCT and SDCT are presented. Except for DSCC, the effect of DCT was significant for the traits investigated (Table 4). Compared to SDCT, quarters under BDCT showed overall more favorable SCS, N_S_, M_S_, L_S_, and PMN_S_. In particular, the LSM of SCS was 1.85 in BDCT and 2.68 in SDCT, corresponding to a SCC around 45,000 and 80,000 cells/mL. After the back-transformation, the LSM of N_S_, M_S_, and L_S_ approximately correspond to 26,538, 15,032, and 15,455 cells/mL for BDCT and to 44,632, 22,162, and 21,857 cells/mL for SDCT. Milk DSCC was not affected by DCT, being similar in BDCT (0.76) and SDCT (0.75).

**Figure 1:**
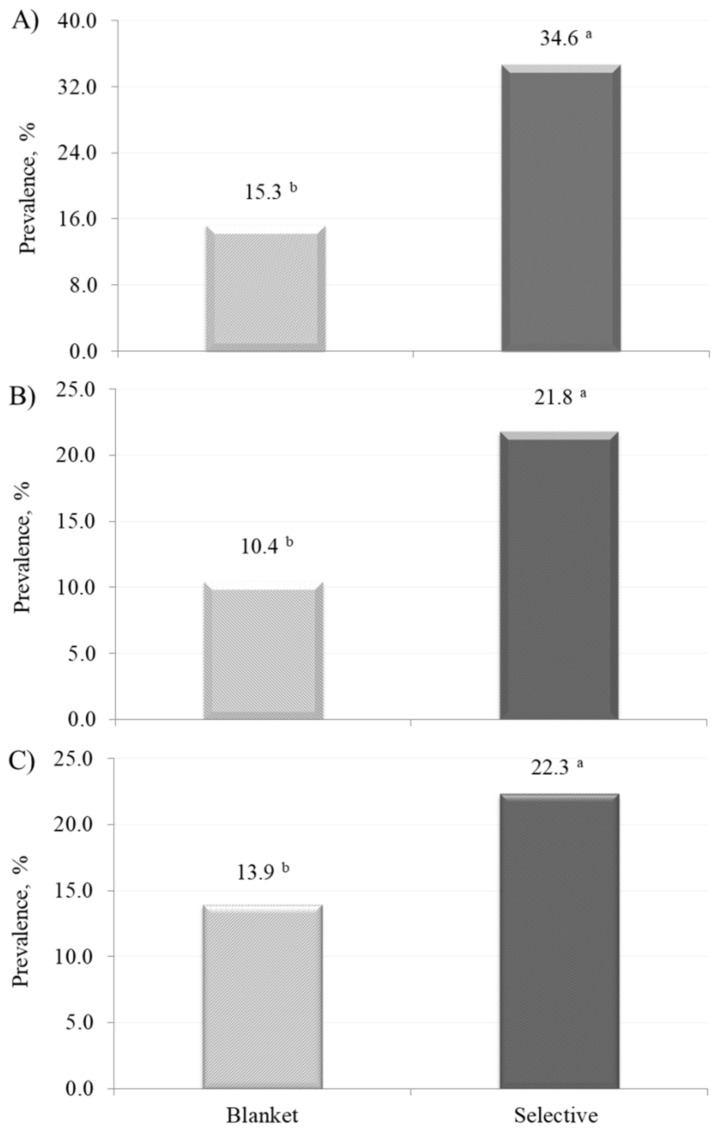
Prevalence of quarters (**A**) with ‘high’ milk somatic cell count (≥100,000 cells/mL), (**B**) with ‘high’ milk polymorphonuclear cells (≥130,000 cells/mL), and (**C**) labelled as ‘positive’ or ‘borderline’ by the device after drying off in blanket and selective dry cow therapy experimental group. Superscript letters indicate significant difference (*p* < 0.05) according to the Bonferroni–Holm step-down adjustment.

The prevalence of ‘high’ SCC differs between experimental groups (Figure 1). In particular, the amount of quarters yielding milk with SCC ≥ 100,000 cells/mL after drying off of SDCT was more than double the one of BDCT (Figure 1). Similarly, the prevalence of ‘high’ PMN (≥130,000 cells/mL) was significantly superior in SDCT than in BDCT, with values of 21.78 and 10.42%, respectively (Figure 1).

Supporting this, the number of quarters classified as ‘borderline’ or ‘positive’ by the QScout^®^ Farm Lab software was greater in SDCT than in BDCT (Figure 1). 

**Figure 2:**
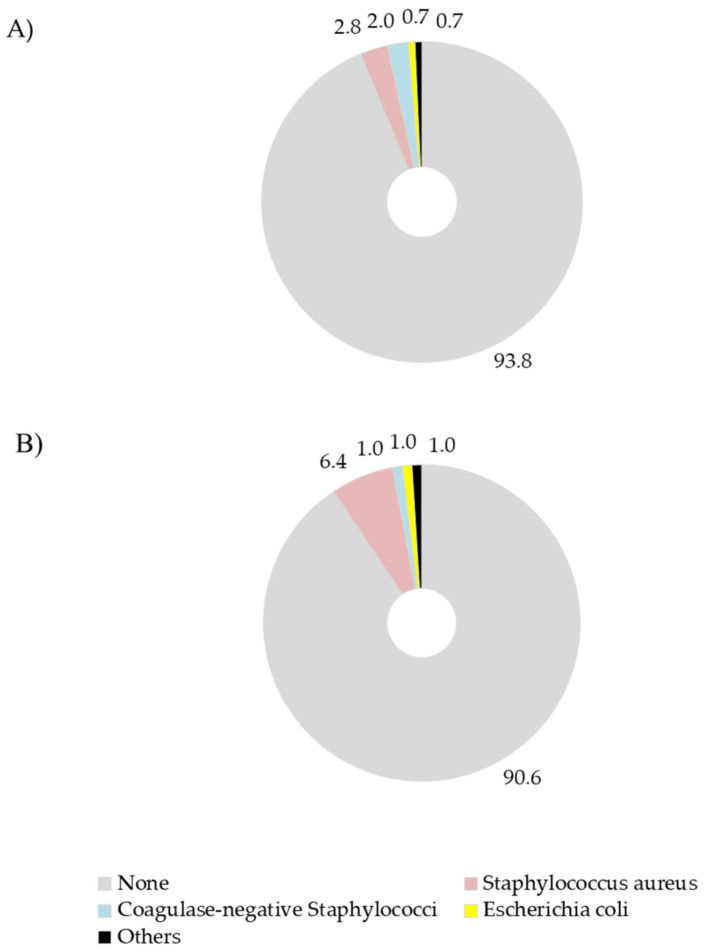
Percentage of quarters showing pathogen(s) for (**A**) blanket and (**B**) selective dry therapy experimental group. Differences between the two groups were not significant (*p* ≥ 0.05) according to the Bonferroni–Holm step-down adjustment.

Microbiological analyses carried out on milk samples after drying off revealed that the 93.8 and the 90.6% of quarters were free of pathogens in BDCT and SDCT, respectively (Figure 2). In both groups pathogens were isolated in a small fraction of quarters and no significant differences were found between the two DCT groups.

Clinical mastitis was diagnosed in 3 and 5 cows within BDCT and SDCT, respectively, representing the 7.32% and the 8.62% of the animals under experimental trial in the two groups. According to the test performed, the two prevalences were similar (*p* = 0.84).

## 4. Discussion

### 4.1. Descriptive Statistics and Correlations

Random allocation allowed to set up comparable groups in terms of milk traits, as traits average and distribution were similar in the two groups before drying off. Nevertheless, M_S_ was an exception. Therefore, to prevent potential biased estimates due to random allocation the analysis of variance included the value of the trait (covariate) recorded before drying off. Milk SCS was greater and more variable after drying off than before, i.e., in early than in late lactation. In fact, SCC is usually very high in colostrum and in milk yielded in the first 2 weeks after calving compared to other stages of lactation. Milk SCC is reported to be rather high immediately after calving, to slightly decrease until 50–60 d in milk, and then to linearly increase up to the end of lactation likely due to a concentration effect [22]. The DSCC was similar in the two samplings (Table 1) within BDCT and within SDCT, suggesting that, regardless of the baseline SCC, the ratio of PMN to SCC was similar before and after drying off. 

Milk SCS was significantly strongly correlated with all the traits investigated, but DSCC (Table 3). Similarly, correlations among the SCC fractions (N_S_, M_S_, L_S_, and PMN_S_) showed overall moderate to strong magnitude. As previously mentioned, part of the correlations obtained for DSCC were negative and/or very low in magnitude, suggesting that this trait per se is weakly associated with an increase in total SCC and in SCC fractions. In fact, the DSCC alone is not able to discriminate between cows with high and low SCC, because a healthy quarter may yield milk with low SCC but at the same time with very high PMN. Moreover quarters with normal level of SCC but high PMN may also not present anomalies in milk, high colony-forming unit pathogen(s) at microbiological test, or signs of inflammation [23].

### 4.2. Fixed Effects

For each trait, the analysis of variance adjusted for the value recorded on the last sampling before dry period. The analysis considered the random effect of the cow nested within the DCT group, therefore the outcomes of the present study were corrected for cow-specific intrinsic factors and/or potential exogenous noises due to permanent environmental conditions. Milk SCS, N_S_, M_S_, L_S_, and PMN_S_ (Table 4) of cows under BDCT showed more favorable LSM compared to those under SDCT. Since only healthy and uninfected quarters were considered for the analysis, a level of SCC largely below the conventional alert threshold [19,20,21] was expected in both groups. Results reported in Table 4 suggest that all SCC fractions are generally affected by the experimental group, with SDCT quarters showing unfavorable and high concentration of N, M, L, and PMN compared to BDCT. The DSCC was similar in the two groups, suggesting that this trait alone seems not an accurate indicator of inflammation, as does not account for the SCC baseline level. For this reason, researchers suggest that DSCC should be coupled with other routinely available milk data, such as SCS, pH, lactose content, and electrical conductivity [23,24,25]. The PMN_S_ proposed in this study seems a more precise indicator of udder health compared to DSCC, as it simultaneously provides information about total SCC and those fractions known to be predominant during mastitis [11,20]. Commonly, suspicious cows or quarters are reported to show milk SCC ≥ 200,000 cells/mL and at the same time DSCC equal or greater than 0.65 [10,20,26]; it derives that a potential accurate PMN threshold approximately corresponds to 130,000 cells/mL and that cows whose milk present PMN above this value might thus be monitored. Nowadays, several devices other than the QScout^®^ Farm Lab (Advanced Animal Diagnostics, Inc., Morrisville, NC, USA) are able to determine milk PMN [20,23].

The significant differences observed for prevalence of ‘high’ SCC (≥100,000 cells/mL), ‘high’ PMN (≥130,000 cells/mL), and suspicious quarters between the two groups (Figure 1) confirmed that BDCT tends to reduce the risk of having elevated SCC in the subsequent lactation compared to SDCT, in agreement with [6]. In other words, BDCT leads to a lower SCC in quarter milk at the onset of lactation by limiting proliferation of pathogens and number of white cells present in the mammary gland alveolar lumen. From a practical point of view, BDCT seems in favor of farmer’s profit whether penalties for high milk SCC or rewards for low SCC are applied by the dairy industry. Nevertheless, this study milk SCC data are exclusively referred to the first sampling after drying off, not to the whole lactation, thus an evaluation of the DCT impact on a 305-d basis was not possible. Numerically, the clinical mastitis prevalence of BDCT (7.32%) was slightly lower than that of SDCT (8.62%) but no significant difference was detected by the statistical test; overall, the frequency of clinical mastitis events recorded from 0 to 305 d in milk in cows under experimental trial was in accordance with other papers reporting prevalence and incidence of udder diseases in dairy cows [3]. 

Apart from this, an extremely low concentration of SCC in milk may also be not optimal from a veterinarian perspective [27,28]. In bovine milk, somatic cells consist in white blood cells in charge of the individual immune response. Immune response could be impaired at udder level if SCC is below a certain physiological threshold, since tissues are more exposed to pathogens infection and proliferation [28].

Finally, in both groups, microbiological analyses revealed that more than 90% of quarters were free of pathogens after drying off (Figure 2). Such finding was expected, since the focus was only put on healthy quarters before drying off. 

As only one commercial farm joined the present experiment, outcomes of this case study cannot be extended to other farming systems, cattle breeds, and countries, as there might be differences related to DCT protocol adopted, antibiotic, genetic resistance to mastitis, hygienic conditions, milking system, and dry period management strategies.

### 4.3. Implications

Although higher SCC and PMN were found in SDCT than in BDCT group at the onset of lactation, BDCT implies use of antibiotics for preventive purpose. For this reason, BDCT might be discouraged if considering the current trends and concerns on antimicrobial use in livestock, ethical aspects, and European Union restrictions that will be implemented in a few months [29]. In fact, antimicrobial use in production animals is a hot-topic that has drawn the attention of international organizations, such as the European Public Health Alliance (EPHA), the Food and Agriculture Organization of the United Nations (FAO), and the European Food Safety Authority (EFSA).

Dairy farmers should be informed of types, benefits and costs related to blanket antibiotic treatment and should be aware of pros and cons of new alternative DCT protocols. In fact, SDCT protocols may considerably differ in terms of efficiency, labor, management, and costs. As an example, the use of teat sealant on all the quarters translates into around EUR 24 per cow for a single dry period. Therefore, moving from traditional BDCT to an alternative DCT can be very easy or very challenging. Given these considerations, it is clear that a unique standard protocol for the best DCT is not feasible and applicable. Each specific context should be carefully evaluated through a cost-benefit analysis by the farmer in order to go for the most appropriate approach that maximizes the profit by compromising pros and cons.

This study demonstrates that PMN_S_ would be more useful than DSCC to monitor udder health. From 2022 the European Union will prohibit the routine use of antimicrobials for preventive scopes in livestock in order to reduce the risk of antimicrobial resistance in both farm animals and human beings. Devices commercially available for milk SCC counting are also able to provide PMN count, so PMN_S_ can be easily derived and then used as an indicator of mammary gland inflammation. This is of particular relevance for future generations of cows, as a more prudent use of antibiotics is going to be mandatory from January 2022 in several European countries [30].

It is important to highlight that the present study does not provide evidence that BDCT is associated with an augmented antimicrobial resistance in dairy cattle. In fact, in general it is rather difficult to demonstrate that preventive and repeated antibiotic treatments in dairy cows have caused an increased antimicrobial resistance in mastitis pathogens. However, scientific community is aware that there is a causal relationship between imprudent use of antibiotic and antimicrobial resistance in livestock and it is public knowledge that the use of certain drugs for preventive scope was not properly monitored at farm level in the past and not subjected to restrictions [30]. According to the traditional routine of commercial farms, BDCT has been successfully used for decades and may have contributed to the restraint of milk SCC, mastitis incidence, udder health issues and veterinary treatments at the onset of lactation. Antimicrobial resistance may be a side collateral effect of BDCT [30], but at the same time SDCT could be more laborious than BDCT and may imply some extra costs, e.g., for sampling and analysis of individual quarters milk (4 per cow) or for laboratory milk analyses of cows out of official milk recording.

## 5. Conclusions

The effect of two DCT on milk total SCC-related traits was investigated in this case study using healthy quarters data. Overall, lower and more favorable LSM were estimated for BDCT than for SDCT. A greater proportion of quarters with ‘high’ SCC (≥100,000 cells/mL) and ‘high’ PMN (≥130,000 cells/mL) was found in cows that underwent SDCT. Overall, outcomes suggest that BDCT reduces the risk of having high SCC levels at the onset of lactation compared to SDCT. On the other hand, the prevalence of clinical mastitis events recorded by the veterinarian was similar in the two groups; the lack of difference in clinical mastitis prevalence suggests that BDCT and SDCT are equally efficient in preventing clinical events in the following lactation. The European legislation is going to put constraints on the routine use of antibiotic for preventive purposes in livestock, consequently, dairy farmers performing BDCT will be asked to adjust their drying off management practices by the end of 2021. Results of this case study demonstrate that farmers should expect an increase in total SCC and in some SCC fractions in healthy quarters when they will move from BDCT to SDCT; however, we cannot conclude that this necessarily translate into an increased mastitis susceptibility or into an impaired resistance to mastitis without further investigations. As soon as BDCT will be forbidden in Europe, milk and health data collected on a large scale in different countries and farming system will definitely allow reliable evaluations and provide more comprehensive answers.

## Figures and Tables

**Table 1:** Overview of data available for blanket (BDCT) and selective dry cow therapy (SDCT) experimental group.

Item	BDCT	SDCT
Number (*n*)		
Overall cows	98	102
Cows after restrictions	41	58
Quarters after restrictions	144	202
Quarters/cow	3.51	3.48
Frequency (%)		
Parity 1	77.6	75.6
Parity 2	15.5	17.1
Later parities (3 + 4)	6.9	7.3
Dry period length (d)		
Average	65.95	63.90
Standard deviation	6.13	9.14

**Table 2:** Post-editing mean of the traits ^1^ in blanket (BDCT) and selective dry cow therapy (SDCT) experimental group. The standard deviation of each trait is given between parentheses.

Trait	Before Drying Off		After Drying Off	
	BDCT	SDCT	BDCT	SDCT
SCS	1.71 (1.08)	1.97 (1.23)	1.82 (1.79)	2.57 (1.93)
N_S_	4.41 (0.96)	4.58 (1.12)	4.75 (1.81)	5.33 (2.09)
M_S_	3.58 (1.04)	3.95 (1.36)	3.84 (1.51)	4.46 (1.71)
L_S_	3.81 (0.98)	4.02 (1.06)	3.95 (1.55)	4.39 (1.52)
PMN_S_	1.37 (1.03)	1.52 (1.18)	1.52 (1.79)	2.14 (2.01)
DSCC	0.78 (0.19)	0.74 (0.20)	0.76 (0.23)	0.75 (0.21)

^1^ SCS = somatic cell score; N_S_ = score of neutrophils count; M_S_ = score of macrophages count; L_S_ = score of lymphocytes count; PMN_S_ = score of polymorphonuclear cell count; DSCC = ratio of polymorphonuclear cell count to total somatic cell count.

**Table 3:** Pearson correlations ^1^ (*p* < 0.05) of milk traits ^2^ within blanket (BDCT) and selective dry cow therapy (SDCT) experimental group after drying off.

Trait	BDCT					SDCT				
	SCS	N_S_	M_S_	L_S_	PMN_S_	SCS	N_S_	M_S_	L_S_	PMN_S_
N_S_	0.95					0.96				
M_S_	0.83	0.72				0.81	0.72			
L_S_	0.89	0.81	0.65			0.85	0.80	0.65		
PMN_S_	0.97	0.97	0.71	0.91		0.97	0.97	0.70	0.88	
DSCC	0.10 ^ns^	0.17	−0.18	0.21	0.23	0.14 ^ns^	0.20 ^ns^	−0.04 ^ns^	0.19	0.19

^1^ ns = not significant; ^2^ SCS = somatic cell score; N_S_ = score of neutrophils count; M_S_ = score of macrophages count; L_S_ = score of lymphocytes count; PMN_S_ = score of polymorphonuclear cell count; DSCC = ratio of polymorphonuclear cell count to total somatic cell count.

**Table 4:** Significance (*p*-value) of the dry cow therapy fixed effect and least squares means with standard error estimated for blanket (BDCT) and selective (SDCT) experimental group. Different superscript letters within trait indicate significantly different least squares means at *p* < 0.05.

Trait ^1^	*p*-Value	BDCT		SDCT	
SCS	0.004	1.85 ^b^	(0.23)	2.68 ^a^	(0.18)
N_S_	0.012	4.73 ^b^	(0.25)	5.48 ^a^	(0.19)
M_S_	0.048	3.91 ^b^	(0.23)	4.47 ^a^	(0.17)
L_S_	0.040	3.95 ^b^	(0.20)	4.45 ^a^	(0.15)
PMN_S_	0.009	1.53 ^b^	(0.23)	2.25 ^a^	(0.17)
DSCC	0.785	0.76 ^a^	(0.03)	0.75 ^a^	(0.02)

^1^ SCS = somatic cell score; N_S_ = score of neutrophils count; M_S_ = score of macrophages count; L_S_ = score of lymphocytes count; PMN_S_ = score of polymorphonuclear cell count; DSCC = ratio of polymorphonuclear cell count to total somatic cell count.

## Data Availability

The data used in the present study are available upon request from the corresponding author.
